# Association of donor and recipient single-nucleotide polymorphisms of interleukin-1 gene with outcomes after allogeneic hematopoietic stem cell transplantation in childhood

**DOI:** 10.1007/s00432-025-06380-x

**Published:** 2025-11-25

**Authors:** Katharina Kämpfner, Susan Wittig, Bernd Gruhn

**Affiliations:** Department of Pediatrics, Jena University Hospital, Am Klinikum 1, D-07747 Jena, Germany

**Keywords:** Interleukin 1, Single-nucleotide polymorphism, Allogeneic hematopoietic stem cell transplantation, Children, Acute graft-versus-host disease

## Abstract

**Purpose:**

Complications such as graft-versus-host disease (GVHD), hepatic sinusoidal obstruction syndrome, and infections compromise the success of allogeneic hematopoietic stem cell transplantation (HSCT) as a treatment modality for malignant or genetic diseases. Identification of beneficial non-human leukocyte antigens (HLA), such as cytokines, is one approach to reduce the rate of unintended events. This study investigated the association between single-nucleotide polymorphisms (SNPs) of the gene of the proinflammatory cytokine interleukin-1 (IL-1) and treatment outcomes after allogeneic HSCT in a pediatric population.

**Methods:**

In our single-center study, we retrospectively analyzed a cohort of 270 children and their respective donors. They underwent allogeneic HSCT for the first time, and their conditioning regimen was myeloablative in all cases. We used polymerase chain reaction to genotype the SNPs of IL-1α rs1800587 (C > T), IL-1β rs16944 (C > T), and IL-1β rs1143627 (C > T). The outcome measures included overall survival (OS), event-free survival (EFS), relapse rate (RR), transplant-related mortality (TRM), and the occurrence of acute or chronic GVHD.

**Results:**

The distribution of IL-1α rs1800587 genotype was as follows: we observed the CC genotype in 124 of 256 recipients (48.4%) and 132 of 270 donors (48.9%). We detected the CT genotype in 115 patients (44.9%) and 114 donors (42.2%) and found the homozygous TT genotype in 17 children (6.6%) and 24 of their donors (8.9%). The distribution of the SNP IL-1α rs1800587 is in Hardy-Weinberg equilibrium. The incidence of moderate or severe acute GVHD was significantly decreased in recipients receiving a donor transplant with the TT genotype (4% (TT) versus 25% (CC/CT); *p* = 0.028). We found no significant SNP IL-1α rs1800587 (C > T) associations for chronic GVHD, RR, TRM, EFS, and OS. For the other genotypes analyzed, IL-1β rs11644 (C > T) and IL-1β rs1143627 (C > T), we also found no significant associations for acute and chronic GVHD, RR, TRM, EFS, and OS, neither in donors nor in recipients. The results of the multivariate analysis revealed a hazard ratio of 0.17 (confidence interval 0.0229-1.27), and a trend that IL-1α rs1800587 could be an independent risk factor for acute GVHD (*p* = 0.084).

**Conclusion:**

Our study identified the donor IL-1α rs1800587 CC/CT genotype as a possible genetic risk factor for developing moderate to severe acute GVHD (grade II - IV) in pediatric patients who underwent allogeneic HSCT. Once confirmed in further studies, these results may influence the donor selection and prophylaxis to decrease the risk of acute GVHD in children.

## Introduction

Allogeneic hematopoietic stem cell transplantation (HSCT) is a curative treatment approach for hematological malignancies, genetic diseases, and severe immunodeficiencies. Despite careful human leukocyte antigen (HLA) matching, HSCT remains associated with a significant morbidity and mortality rate. This is critically caused by infection, hepatic sinusoidal obstruction syndrome, relapse, and graft-versus-host disease (GVHD). Therefore, single-nucleotide polymorphisms (SNPs) of non-HLA-associated factors, such as cytokines and their receptors, are considered contributing components.

Interleukin 1 (IL-1) is the generic term for a group of molecules that includes ten receptors and eleven cytokines. They are classified according to their primary mode of action into agonistic cytokines (IL-1α, IL-1β, IL-18, IL-33, IL-36α, IL-36β, and IL-36γ), antagonistic receptors (IL-1Ra and IL-36Ra), and anti-inflammatory mediators (IL-37 and IL-38).

Our study focused on the two biologically active forms of IL-1: IL-1α and IL-1β. Their genes are 430 kb wide and located on the long arm of chromosome 2 (Nicklin et al. 2002).

IL-1α is a molecule ubiquitously present in mesenchymal cells and increasingly formed during inflammatory stimulation. Its effect is primarily at the local level. IL-1α is an alarmin, a molecule that signals the loss of membrane integrity of a cell to surrounding cells (Rider et al. 2017). IL-1α acts as a gene expression-enhancing transcription factor (e.g., IL-8) in the nucleus (Werman et al. 2004). As a membrane protein, it also initiates inflammatory signaling pathways (Di Paolo and Shayakhmetov 2016).

Myeloid cells produce IL-1β, which is only detectable during inflammatory metabolism. IL-1α induces the production of IL-1β. IL-1β introduces a proinflammatory response, which is characterized by the synthesis of cytokines and prostaglandins, vasodilation, and immune cell binding to correspondingly activated epithelial molecules. IL-1β causes the transcription induction of crucial factors, which unfold the effects described below by reaction cascades (Dinarello 2009).

Acute and chronic GVHD are distinguished by their manifestations (de novo, in patients with previously cured acute GVHD, or transition from acute GVHD to chronic GVHD). (Filipovich et al. 2005). The severity of damage to the gut, skin, and liver determines the classification of acute GVHD. (Glucksberg et al. 1974; Harris et al. 2016). The chronic GVHD is diagnosed and classified based on the severity of 8 organs (skin, mouth, eyes, gastrointestinal (GI) tract, liver, lungs, joints and fascia, genital tract) (Jagasia et al. 2015).

The pathophysiological processes of acute GVHD begin in the GI tract. The tissue-damaging mechanisms of the conditioning regimen activate innate immune system of the recipient, especially neutrophil granulocytes and monocytes (Reinhardt et al. 2014; Schwab et al. 2014). These cells destroy tight junctions by releasing reactive oxygen species. This process increases the risk that bacteria, fungi, and viruses will enter the intestinal tissue and the bloodstream. These pathogen-associated molecular patterns activate antigen-presenting cells. At the same time, damage-associated molecular patterns (adenosine triphosphate and uric acid) trigger the chemokine and cytokine cascade (e.g., IL-1 folds into IL-1β) (de Mooij et al. 2017; Jankovic et al. 2013). This activates T cells and initiates their proliferation. These cells mediate damage to the target organs, the liver, skin, and GI tract, through corresponding inflammatory reactions (Glucksberg et al. 1974; Zeiser 2020).

The pathophysiological processes of chronic GVHD are still largely unclear. IL-17 mediates fibrotic remodeling of multiple organs such as the skin, liver, and lungs (Varelias et al. 2015). The primary drivers of this response are allo- and autoimmune processes that result from the lack of thymic selection of donor T cells and unselected antibody-producing B cells (Sarantopoulos and Ritz 2015; Socie and Ritz 2014).

To date, 148 SNPs are known for the 31 kDa IL-1α gene, comprising six introns and seven exons (Khazim et al. 2018). The SNP of IL-1α rs1800587 located in the promoter region shows a substitution from the wild-type deoxyribonucleic acid (DNA) base cytosine (C) to thymine (T). The altered SNPs are associated with Alzheimer’s disease (Du et al. 2000; Nicoll et al. 2000), increased tumor risk (Cheng et al. 2014), and autoimmune reactions such as chronic GVHD (Cullup et al. 2003).

IL-1β is a 7.5-kb gene that contains seven exons (Behzadi et al. 2022). To date, IL-1β consists of 144 SNPs (Khazim et al. 2018). IL-1β rs16944 and IL-1β rs1143627, the SNPs we investigated, are located in the promoter region. IL-1β rs16944 is characterized by a base exchange from C to T. It is in a regulatory gene motif that mediates the interaction with transcription factors and an increased activity of the IL-1β promoter. This process has been demonstrated molecularly in patients with bladder, colon, breast, and lung cancer (Landvik et al. 2012). In addition, these patients are more susceptible to infections, e.g., with *Leishmania guyanensis* (da Silva et al. 2019).

IL-1β rs1143627 consists of a base change from T to C. This has been associated with a higher risk of lung cancer (Kiyohara et al. 2010) and chronic periodontitis (Deng et al. 2013). However, the precise mechanism of this SNP remains unclear due to inconsistent study findings (Smith and Humphries 2009).

This study aimed to analyze the association between SNPs of the IL-1 gene (IL-1α rs1800587, IL-1β rs16944, and IL-1β rs1143627) and treatment outcomes in a pediatric population undergoing allogeneic HSCT. We hope our findings can contribute to understanding treatment outcomes in pediatric care.

## Materials and methods

### Patients

We retrospectively analyzed 270 recipients and their matched donors of allogeneic HSCT at the Department of Pediatrics, Jena University Hospital, Jena, Germany, from 1990 to 2019. We excluded patients older than 18 years and children receiving a second or further HSCT.

All subjects gave informed consent, and the study was approved by the Ethics Committee of the Jena University Hospital (2021–2084). The characteristics of the patients are listed in Table 1.

**Table 1:** Characteristics of patients and donors (*n* = 270)

Characteristics	Total, no. (%)
Median age of the patients (y)	9
Median Follow-up (months)	46
Sex of patients	
Male	160 (59.3)
Female	110 (40.7)
Disease	
ALL	89 (33.0)
AML	63 (23.3)
CML	10 (3.7)
JMML	9 (3.3)
EWS/ NBL/ RMS	11 (4.1)
Genetic disease	41 (15.2)
Myelodysplastic syndrome	29 (10.7)
Lymphoma	7 (2.6)
Severe aplastic anemia	11 (4.1)
Conditioning regimen (based on)	
Total body irradiation	93 (34.4)
Busulfan	126 (46.7)
Treosulfan	18 (6.7)
Cyclophosphamide	10 (3.7)
Thiotepa and 5-Fluorouracil	21(7.8)
Others	2 (0.7)
GVHD prophylaxis	
Cyclosporin A and methotrexate	148 (54.8)
Cyclosporin A	55 (20.4)
Cyclosporin A and mycophenolate mofetil	17 (6.3)
Mycophenolate mofetil	11 (4.1)
Methotrexate	1(0.37)
None	38 (14.1)
Antithymocyte globulin in addition	171 (63.3)
Acute GVHD	
Total	120 (44.4)
Grade II - grade IV	63 (23.3)
Grade III- grade IV	19 (7.0)
Chronic GVHD	
Total	38 (14.1)
Donor type	
HLA-matched unrelated	127 (47.0)
HLA-mismatched unrelated	36 (13.3)
HLA-identical related	74 (27.4)
HLA-haploidentical related	33 (12.2)
Stem cell source	
Bone marrow	178 (65.9)
Peripheral blood stem cells	90 (33.3)
Umbilical cord blood	2 (0.7)
*ALL* acute lymphoblastic leukemia; *AML* acute myeloid leukemia; *CML* chronic myeloid leukemia; *EWS* Ewing sarcoma; *HLA* human leukocyte antigen; *JMML* juvenile myelomonocytic leukemia; *NBL* neuroblastoma; *RMS* rhabdomyosarcoma	

### Genotyping of the interleukin-1 polymorphism

We isolated the mononuclear cells from the samples (peripheral and cord blood, bone marrow aspirates) and purified them using Ficoll-Hypaque (Sigma, St. Louis, MO, USA). We utilized liquid nitrogen at -196 °C for cryopreservation of the mononuclear cells. We extracted the DNA from these samples using the High Pure PCR Template Preparation Kit (Roche, Mannheim, Germany) following the manufacturer’s instructions. We photometrically quantified the purified template DNA at 260 nm and 280 nm using the BioPhotometer Plus (Eppendorf, Wesseling-Berzdorf, Germany). We combined 1 µL (10 ng/µL) of DNA, 10 µL of genotyping master mix, 9.5 µL of sterile water, and 0.5 µL of primer-probe mix (TaqMan genotyping SNP assays from Applied Biosystems (Foster City, CA, USA)). Samples and five negative controls were transferred into barcoded 96-well optical reaction plates using pipettes. We utilized the 7900HT Fast Real-Time PCR System from Applied Biosystems (Waltham, Massachusetts, USA), equipped with two different detectors, to quantify the DNA. We performed PCR as follows: heating at 95 °C for 10 min, followed by 40 cycles of denaturation at 92 °C for 15 s, annealing and extension at 60 °C for 60 s. We identified the respective SNP using allelic discrimination post-read run. Three different SNPs from the IL-1 gene group were analyzed: IL-1α rs1800587, IL-1β rs16944, and IL-1β rs1143627.

### Definition of clinical endpoints

We classified the patients in accordance with the Mount Sinai Acute GVHD International Consortium (Harris et al. 2016), depending on the severity of acute GVHD in the intestine, liver, and skin. We followed the National Institutes of Health consensus group’s classification of the severity of chronic GVHD according to the assessment of the affected organs. (Filipovich et al. 2005; Harris et al. 2016; Jagasia et al. 2015; Kitko et al. 2021). Transplant-related mortality (TRM) included all deaths without evidence of prior progression or relapse after HSCT. We considered any relapse of the underlying disease as relapse incidence. Relapse risk (RR) is the cumulative relapse incidence rate after HSCT. We defined overall survival (OS) as time from HSCT to death from any cause. We determined event-free survival (EFS) as the time from HSCT to relapse, underlying disease progression, second malignancy, or death.

### Statistical analysis

We tested each SNP for Hardy-Weinberg equilibrium and compared the results using the chi-squared test. The cumulative incidence rates of acute and chronic GVHD were calculated, with death treated as the competing risk. We employed the Kaplan-Meier method to analyze overall survival (OS) and event-free survival (EFS), and we compared the results using the log-rank test. We determined RR, TRM, acute GVHD, and chronic GVHD using the survival calculation with competing risks and the Gray test to determine possible comparative differences (Gray 1988; Scrucca et al. 2007, 2010). For multivariate analysis, we employed the semiparametric proportional hazards model. (Fine and Gray 1999; Scrucca et al. 2010). We conducted the statistical analysis using SPSS 26 (IBM Corporation, Armonk, NY, USA) and R (Foundation for Statistical Computing, 4.0, Vienna, Austria). We considered a p-value below 0.05 as statistically significant and below 0.1 as a trend.

## Results

### Analysis of polymorphisms

Our analysis covered 270 donors and 256 patients for the IL-1α rs1800587 SNP. We examined the IL-1β rs1143627 polymorphism in 270 donors and 255 recipients. Additionally, we checked the IL-1β rs16944 SNP in 257 recipients and 270 donors. The CC genotype of IL-1α rs1800587 was present in 124 recipients (48.4%). One hundred fifteen recipients (44.9%) had the CT genotype, and 17 recipients (6.6%) had the TT genotype. Fourteen cell samples were not analyzable. The distribution of genotypes for the SNP IL-1α rs1800587 among donors was as follows: 132 donors had the CC genotype (48.9%), 114 donors had the CT genotype (42.2%), and 24 donors had the TT genotype (8.9%). We could confirm that the allele distribution of the IL-1α rs1800587 SNP in the donors (*p* = 0.9307) and the recipients (*p* = 0.1562) is consistent with the Hardy-Weinberg equilibrium.

Thirty-five recipients (13.7%) had the CC genotype of the IL-1β rs1143627 polymorphism, 121 patients (47.5%) had the CT genotype, and the TT genotype was found in 99 cases (38.8%). In 15 cases, we were unable to determine the genotype. Thirty-three donors (12.2%) were homozygous for the IL-1β rs1143627 CC genotype, and 110 donors (40.7%) were homozygous for the TT genotype. The heterozygous CT genotype occurred in 127 donors (47.0%). We observe that the SNP IL-1β rs1143627 conforms to the Hardy-Weinberg equilibrium in both donors (*p* = 0.693) and recipients (*p* = 0.8378).

We observed the CC genotype of the IL-1β rs16944 polymorphism in 107 patients (41.6%), the CT genotype in 116 patients (45.1%), and the TT genotype in 34 patients (13.2%). DNA analysis was unsuccessful in 13 cases. Genotyping of the donor samples revealed the IL-1β rs16944 CC genotype in 108 cases (40.0%), the CT genotype in 129 donors (47.8%), and the TT genotype in 33 cases (12.2%). The SNP IL-1β rs16944 is in Hardy-Weinberg equilibrium in both recipients (*p* = 0.7723) and donors (*p* = 0.5602).

In Table 2, we provide a detailed description of the distribution of IL-1α rs1800587 (C > T), IL-1β rs1143627 (C > T), and IL-1β rs16944 (C > T) genotypes. Our distribution of the SNPs IL-1α rs1800587 and IL-1β rs16944 closely matches the literature. (MacMillan et al. 2003).

**Table 2:** Patients and donors’ frequencies of the genotypes for IL1α rs1800587, IL-1β rs1143627, and IL-1β rs16944 SNPs

SNP	Patients No. (%)	Donors No. (%)
IL-1α rs1800587		
Total	256	270
Genotype CC	124 (48.4)	132 (48.9)
Genotype CT	115 (44.9)	114 (42.2)
Genotype TT	17 (6.6)	24 (8.9)
IL-1β rs1143627		
Total	255	270
Genotype CC	35 (13.7)	33 (12.2)
Genotype CT	121 (47.5)	127 (47.0)
Genotype TT	99 (38.8)	110 (40.7)
IL-1β rs16944		
Total	257	270
Genotype CC	107 (41.6)	108 (40.0)
Genotype CT	116 (45.1)	129 (47.8)
Genotype TT	34 (13.2)	33 (12.2)

### Incidence of events

Seventy-five children (27.8%) suffered from relapse. One hundred and twenty children (44.4%) died of relapse (*n* = 41, 15.2%), acute or chronic GVHD (*n* = 11, 4.1%), infection (*n* = 17, 6.3%), or other causes of death (*n* = 51, 18.9%).

### Incidence of graft-versus-host disease

One hundred and twenty patients (44.4%) developed acute GVHD. Sixty-three patients (23.3%) suffered from moderate to severe acute GVHD (grade II, *n* = 44 (16.3%), grade III, *n* = 6 (2.2%), grade IV, *n* = 13 (4.8%)). The median time to onset of GVHD was 28 days.

In addition, 38 recipients (14.1%) developed chronic GVHD. Our findings showed a median onset time of 164 days for chronic GVHD.

### Genetic association with acute graft-versus-host disease

**Fig. 1:**
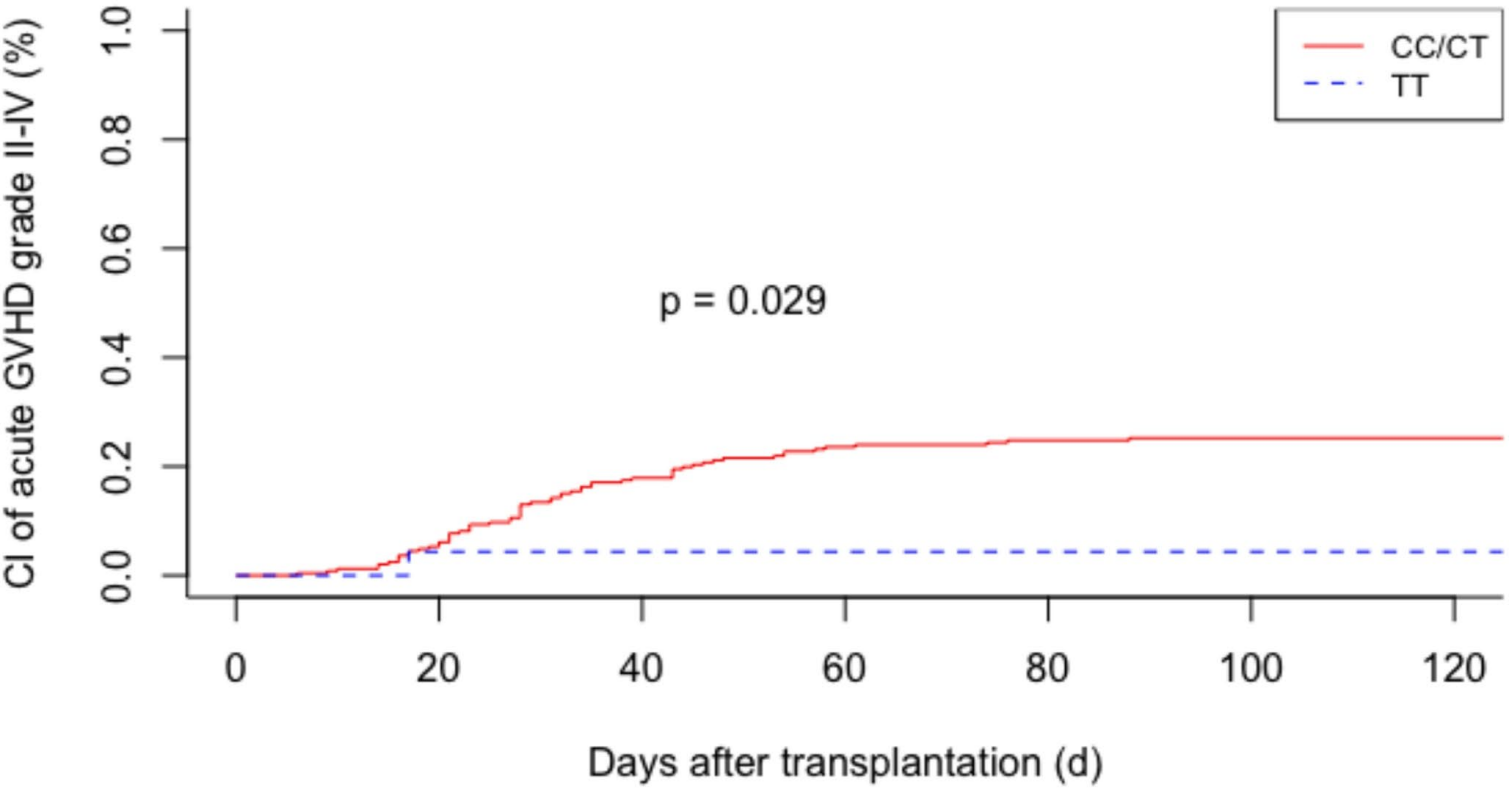
Cumulative incidence (CI) curves summarize the incidence of moderate and severe (grade II to grade IV) acute GVHD in patients with donor IL-1 genotyped for IL-1α rs1800587 (C > T)

To analyze an association between the genotypes IL-1α rs1800587, IL-1β rs16944, and IL-1β rs1143627 of recipients and donors and the occurrence of acute GVHD, we compared those who experienced clinically significant acute GVHD. We found a significantly increased incidence of moderate to severe acute GVHD (grade II - IV) if the patient received a transplant from a donor with the IL-1α rs1800587 CC/CT genotype (25% (CC/CT) versus 4% (TT); *p* = 0.028, Fig. 1). No significant result was found from analyzing the IL-1α rs1800587 patient SNP. IL-1β SNPs showed no significant association between acute GVHD and either donor or recipient genotype.

### Multivariate analysis

We performed a multivariate analysis to assess the potential independent influence of the IL-1α rs1800587 factor on the incidence of acute GVHD after HSCT. We compared the SNP IL-1α rs1800587 with the following possible confounding clinical characteristics: sex, stem cell source, GVHD prophylaxis with antithymocyte globulin, donor-recipient gender match, and HLA match. We observed a trend (*p* = 0.084), suggesting that the CC/CT genotype of IL-1α rs1800587 might be an independent factor for developing acute GVHD. Table 3 shows the results of the multivariate analysis of IL-1α rs1800587.

**Table 3:** Multivariate analysis of donor IL-1α rs1800587

Variable	Acute GVHD	
	HR (95% CI)	p-value
Donor IL-1α rs1800587 (TT versus CC/CT)	0.17 (0.0229–1.27)	0.084
Female sex	1.204 (0.6953–1.97)	0.550
Stem cell source (BM vs. PBSC)	0.738 (0.4352–1.36)	0.370
GVHD prophylaxis with ATG	0.8785 (0.7546–2.09)	0.380
Gender match	1.023 (0.6168–1.70)	0.930
HLA match	1.324 (0.7578–2.38)	0.310
*ATG* antithymocyte globulin; *BM* bone marrow; *CI* confidence interval; *GVHD* graft-versus-host disease; *HLA* human-leukocyte antigen, *HR* hazard ratio; *PBSC* peripheral blood stem cells		

### Other endpoints

We also investigated the incidence of chronic GVHD, OS, EFS, RR, and TRM, as well as the possible influence of the SNPs of IL-1α rs1800587, IL-1β rs1143627, and IL-1β rs16944. We found no effect of the investigated IL-1 polymorphisms on chronic GVHD. In the children’s OS, we observed no significant correlation among the analyzed SNPs. The results revealed no significant association between the SNPs and EFS. Our study demonstrated that RR and TRM were not associated with the IL-1 polymorphisms.

## Discussion

HSCT is a curative treatment option for various diseases, such as malignant tumors and genetic or metabolic disorders. Although HLA matching of recipient and donor is standardized, multiple complications such as GVHD, infections, or hepatic sinusoidal obstruction syndrome are still a challenge in the treatment of these patients (Hierlmeier et al. 2018). Therefore, we focused on non-HLA polymorphisms as a possible influencing factor. SNPs in the gene of the inflammatory cytokine IL-1, which plays a significant role in acute GVHD, may be key to developing complications. (de Mooij et al. 2017). Our study aimed to analyze the association between the IL-1α rs1800587 SNP and the IL-1β SNPs rs16944 and rs1143627 and the outcome of HSCT in a pediatric cohort.

Our analyses have shown that patients who receive cells from a donor with the CC or CT genotype of the IL-1α rs1800587 SNP have a higher rate of acute GVHD (grade II to IV). Our multivariate analysis indicated a trend, suggesting that the IL-1α rs1800587 CC/CT genotype might be an independent risk factor for developing acute GVHD. These results are partly consistent with a cohort of patients with leukemia (*n* = 57) in which the effects were detected only for the CC genotype. They report an Odds Ratio of 3.49 (1.20-10.12) and a p-value of 0.02 (Noori-Daloii et al. 2013). Other analyses involving siblings or unrelated donors did not yield any significant results, indicating that the IL-1α rs1800587 SNP causes a higher incidence of acute GVHD (Cullup et al. 2003; MacMillan et al. 2003; Mehta et al. 2007).

The SNP IL-1α rs1800587 exhibits a point mutation from C to T. The TT genotype creates a new consensus site to which the transcription factor Skn-1 can bind. IL-1α SNP rs1800587, as part of the promoter region of IL-1α, influences the expression of IL-1α and, correspondingly, IL-1β.

Nowadays, IL-1α is mainly recognized for its proinflammatory role, which suggests that the occurrence of acute GVHD or organ damage from it should decrease if patients receive IL-1 RA as a natural antagonist of IL-1α. However, studies using recombinant or human IL-1 receptor antagonists have shown inconsistent results. In studies by (Antin et al. 1994) and (McCarthy et al. 1996), the incidence of severe acute GVHD was reduced. In contrast, a randomized controlled trial by (Antin et al. 2002) indicated that administration of the recombinant receptor antagonist did not affect the incidence of acute GVHD. Whether these findings are attributable to the still novel administration regimen or to the biology of IL-1α remains unclear. Further research is needed.

Several intracellular mechanisms are known to limit IL-1α’s proinflammatory effects, indicating that IL-1α’s proinflammatory action depends on the cell’s metabolic context. When cells undergo necrosis, IL-1α has a clear proinflammatory effect. However, during apoptosis, IL-1α is transferred from the cytosol to the cell nucleus and does not trigger inflammation by binding to chromatin (Dinarello 2018). Whether these processes contribute to our results should be explored in further research.

We found no significant correlation between IL-1α rs1800587, independent of donor or recipient, and OS, EFS, RR, TRM, and chronic GVHD. These findings align with the results of one study. (Mehta et al. 2007). Some studies have described patients who did not have the TT allele at IL-1α rs1800587 having a higher rate of chronic GVHD (Cullup et al. 2003; MacMillan et al. 2003). Other results (MacMillan et al. 2003) showed an increased OS for the IL-1α rs1800587 with the TT genotype.

The analyses of OS, acute or chronic GVHD, TRM, RR, and EFS showed no correlation with the SNPs of IL-1β rs1143627 and IL-1β rs16944. Several studies also found no influence of these SNPs (Cullup et al. 2003; Dickinson et al. 2001; Elbahlawan et al. 2012). Hyvarinen et al. (2017) reported a higher rate of chronic GVHD. MacMillan et al. (2003) also found a higher OS rate when the genotype had at least one T in either the donor or recipient. We expected identical results in the statistical analyses for the SNPs of IL-1β rs16944 and IL-1β rs1143627, as these SNPs are located in regions with a highly conserved genotype and a 99.5% linkage disequilibrium (Cullup et al. 2001; Read et al. 2000). Our results confirmed this assumption.

Several factors might account for the differences between our results and those of other researchers. The cohorts are often highly heterogeneous in terms of clinical standards (stem cell source, conditioning regimen, and GVHD prevention) and patient-related factors (donor/recipient age, graft gender mismatch, diagnosis, stage, and ethnic background of donor/recipient, among others). The statistical approaches are only partly comparable. Unlike other studies that included adults and children, we analyzed a pediatric cohort exclusively.

The data situation is similarly unclear for other SNPs of non-HLA-associated factors: NOD2, IL-17, IL-23 receptor (IL-23R), and macrophage migration inhibitory factor (MIF). All these factors are also involved in the pathophysiology of acute GVHD through various mechanisms, including regulating inflammation (NOD2), promoting inflammation (IL-17), inducing proliferation and interferon-γ (IL-23R), and amplifying and prolonging immune responses (MIF). SNPs of NOD2 have been linked to increased severe acute GVHD in one study (Holler et al. 2004), while another study found no effect on acute GVHD. (Gruhn et al. 2009) SNPs in the IL-17 gene are linked to a higher risk of acute GVHD (Espinoza et al. 2011a, b). One study found that the effect of SNPs in the IL-23R region was insignificant (Nguyen et al. 2010). In contrast, Gruhn et al. (2009) observed a reduced incidence of acute GVHD and an SNP of IL-23R. SNPs of MIF are associated with an increased rate of acute GVHD. (Aharon et al. 2025)

The numerous genetic links identified between non-HLA SNPs and acute GVHD occurrence after HSCT, have so far only been investigated in research settings. To date, no non-HLA SNPs have been incorporated into guidelines for donor selection or GVHD prophylaxis, nor has it been implemented in clinical practice.

The present analyses enhance the extensive knowledge concerning non-HLA polymorphisms. Our findings suggest that IL-1α rs1800587 in the TT genotype of the donor may contribute to a reduced rate of acute GVHD, which could be helpful in selecting potential donors and for specified GVHD prophylaxis. However, the applicability of our results is limited by the single-center design and retrospective nature of the study. The multivariate analysis also showed a trend, but further research in larger, more homogeneous studies is necessary. Additionally, the current data on IL-1 blockade are inconsistent.

Therefore, we recommend conducting further studies to test and potentially confirm our hypothesis that patients receiving stem cells from donors with the TT genotype in IL-1α rs1800587 have a lower rate of acute GVHD.

## Data Availability

No datasets were generated or analysed during the current study.
